# Potential Application of Yeast Cell Wall Biopolymers as Probiotic Encapsulants

**DOI:** 10.3390/polym15163481

**Published:** 2023-08-20

**Authors:** Gemilang Lara Utama, Lidya Oktaviani, Roostita Lobo Balia, Tita Rialita

**Affiliations:** 1 Faculty of Agro-Industrial Technology, Universitas Padjadjaran, Jalan Raya Bandung-Sumedang Km. 21, Jatinangor, Sumedang 45363, Indonesia; lidya17005@mail.unpad.ac.id (L.O.); tita.rialita@unpad.ac.id (T.R.); 2 Center for Environment and Sustainability Science, Universitas Padjadjaran, Jalan Sekeloa Selatan 1 No 1, Bandung 40134, Indonesia; 3 Veterinary Study Program, Faculty of Medicine, Universitas Padjadjaran, Jalan Raya Bandung-Sumedang Km. 21, Jatinangor, Sumedang 45363, Indonesia; roostita.balia@unpad.ac.id

**Keywords:** β-glucans, mannoproteins, chitin, biopolymers, encapsulant

## Abstract

Biopolymers of yeast cell walls, such as β-glucan, mannoprotein, and chitin, may serve as viable encapsulants for probiotics. Due to its thermal stability, β-glucan is a suitable cryoprotectant for probiotic microorganisms during freeze-drying. Mannoprotein has been shown to increase the adhesion of probiotic microorganisms to intestinal epithelial cells. Typically, chitin is utilized in the form of its derivatives, particularly chitosan, which is derived via deacetylation. Brewery waste has shown potential as a source of β-glucan that can be optimally extracted through thermolysis and sonication to yield up to 14% β-glucan, which can then be processed with protease and spray drying to achieve utmost purity. While laminarinase and sodium deodecyle sulfate were used to isolate and extract mannoproteins and glucanase was used to purify them, hexadecyltrimethylammonium bromide precipitation was used to improve the amount of purified mannoproteins to 7.25 percent. The maximum chitin yield of 2.4% was attained by continuing the acid–alkali reaction procedure, which was then followed by dialysis and lyophilization. Separation and purification of yeast cell wall biopolymers via diethylaminoethyl (DEAE) anion exchange chromatography can be used to increase the purity of β-glucan, whose purity in turn can also be increased using concanavalin-A chromatography based on the glucan/mannan ratio. In the meantime, mannoproteins can be purified via affinity chromatography that can be combined with zymolase treatment. Then, dialysis can be continued to obtain chitin with high purity. β-glucans, mannoproteins, and chitosan-derived yeast cell walls have been shown to promote the survival of probiotic microorganisms in the digestive tract. In addition, the prebiotic activity of β-glucans and mannoproteins can combine with microorganisms to form synbiotics.

## 1. Introduction

Encapsulation is a process that encases active substances in a protective shell, improving their stability, controlled release, and targeted distribution. Yeast cell wall biopolymers have attracted interest in recent years as viable materials for encapsulation. Yeast particles are hollow and porous microspheres that are produced as leftovers during the synthesis of food-grade yeast extracts. The versatility of encapsulation with yeast cell wall biopolymers makes it suitable for a wide range of applications. In agriculture, encapsulated biopesticides can be precisely delivered to target pests, reducing environmental impact and improving efficacy. In the food industry, encapsulated flavors and fragrances are used for enhancing the sensory properties of food products. Additionally, encapsulated beneficial microorganisms such as probiotics can be employed to improve the delivery systems, ensuring better viability and controlled release. 

Probiotic encapsulation is a process that involves wrapping probiotic cells in an encapsulant matrix to preserve them from environmental degradation and release them at controlled levels under specific circumstances [1]. Encapsulation can help probiotics overcome their environmental resistances problems. In order for the core substance to move through the digestive tract without harming cells, it must be shielded from external factors such as oxygen, strong acids, and stomach conditions via encapsulation [2].

The compatibility of all the components, including the probiotic microbe strain, the encapsulation technique, and the encapsulant material employed, determines how effective the protection offered by encapsulation is. A microcapsule is made up of a nucleus with a very small diameter that can range from a few micrometers to one millimeter in size and is encased in a spherical, tough, semipermeable membrane [3]. The use of encapsulant materials in food applications is generally regarded as safe.

It is crucial to choose the right biopolymer for encapsulant since it affects how well it protects cells. Biocompatible, biodegradable, processable, and probiotic-neutral biopolymers can be employed to encapsulate probiotics [4]. Proteins, polysaccharides, and lipids make up majority of the encapsulant materials used in probiotic encapsulation today. Examples of protein biopolymers include zein, soy protein, collagen, and gelatin. Polysaccharides include cellulose derivatives, starch, alginate, and chitosan. These naturally occurring biopolymers, or chemically altered versions of them, are frequently applied in different probiotic encapsulation techniques as single layer coatings or in combination to build structural coating layers [5].

The components of the yeast cell wall have potential as novel encapsulation substance as a probiotic microencapsulant for bacteria [6]. The polysaccharides and glycoproteins that make up the yeast cell wall are its primary building blocks. One commonly used type of yeasts, *Saccharomyces cerevisiae*, has a cell wall with the potential for encapsulation that is 100–200 nm thick and is composed of β-glucan polymers, which comprise glucose monomers containing β-1,3-glucan and β-1,6-glucan bonds together, mannan (a polymer of mannose), which pairs with proteins to form mannoproteins, and a small amount of chitin. A hydrophilic environment and cell porosity are provided by the mannoprotein layer on the surface of the yeast cells, and the cell wall’s form and stability are preserved by β-glucan [7]. Chitin is a substance that provides the interior of cells with their strength, rigidity, and defense, while a plasma membrane, selectively permeable and made up of a layer of phospholipid bilayer, is also part of the cell wall [6].

Whether the yeast cells are alive or not, plasmolyzed partially or entirely, or both, they can be employed as microcapsules [8,9]. Probiotic bacteria that had been previously encapsulated with calcium alginate, so that they had bigger cell sizes compared to yeast cells themselves, were successfully microencapsulated using cells from *S. cerevisiae* as encapsulants [10]. The inclusion of a layer of yeast cells in digestive juices with a triple-layered coating of alginate polymers improves the durability of probiotic bacteria while delaying gastric juice absorption into the microcapsules [11].

The yeast cell wall protects the encapsulated molecule against moisture, high temperature, light, oxygen, and evaporation, ensuring stability [12]. The cell wall of *S. cerevisiae* protects curcumin compounds from degradation at high temperatures (150–200 °C) better than β-cyclxodextrin and modified starch [13]. β-glucan from spent brewer’s yeast cells can protect probiotic Lactobacillus bacteria after freeze-drying [14]. Spray-dried sunflower oil covered with spent brewer’s yeast cells was oxidatively stable [15]. Mannoproteins and β-glucans extracted from yeast cell walls have antioxidant capabilities and increase oxidative stability [16]. Mannoprotein component extracted from yeast cell walls stimulated the growth of probiotic bacteria *L. plantarum* and *L. salivarius* in gastrointestinal fluids and allowed them to colonize the intestine [17]. Chitin is present in modest amounts, together with β-glucan polymers and mannoprotein polymers. Deacetylation of chitin yields chitosan, a popular coating material. Chitin to chitosan conversion makes the polymer more soluble and usable [18]. Chitosan coating can help probiotic bacteria survive storage and digestion [19].

It has been known that the yeast cell walls materials such as β-glucan, mannoprotein and chitosan are Generally Recognized as Safe (GRAS). Because of its unique composition and structure, yeast cell walls and their contents have shown promise as a promising encapsulation matrix for probiotic microorganisms. Based on this description, this literature review will determine the possibility of biopolymers extracted from yeast cell walls as novel encapsulant materials to enhance the survival of probiotics in the digestive system.

## 2. Yeast Cell Walls: Structures and Compositions

Yeast cells have been shown to successfully encapsulate various food compounds. Several yeast species, including *Saccharomyces cerevisiae*, *Saccharomyces bayanus*, *Candida utilis*, *Kluyveromyces fragilis*, *Torulopsis lipofera*, *Endomyces vernalis*, and *Cryptococcus curvatus*, have been used as microencapsulants [6].

**Figure 1:**
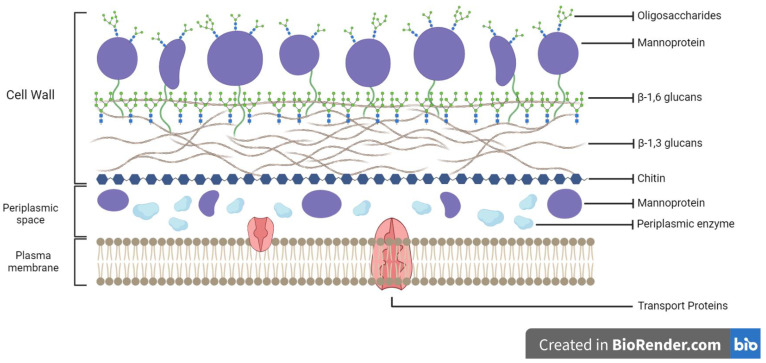
The structure of the yeast cell wall.

The yeast cell wall is responsible for the yeast’s encapsulation capacity. The characteristics of the yeast cell wall affect the interaction between cells and encapsulated substances or components. Therefore, understanding the nature of the yeast cell wall can improve comprehension of the microencapsulation process. Around 15–20% of the yeast cell’s dry bulk is composed of the cell wall. The thickness of the yeast cell wall varies considerably, from 70 to 200 nm. The thickness of the yeast cell walls increases in response to osmotic pressure or compression. The cell wall of yeasts is a highly polar, double-layered matrix. The yeast cell wall is composed primarily of glycoproteins and polysaccharides. It has a network of β-1,3-glucan that is cross-linked with β-1,6-glucan, a mannoprotein layer, and chitin [20]. The structure of the yeast cell wall is depicted in Figure 1.

β-glucan is the primary polysaccharide of the yeast cell wall, which consists of a network of β-1,6-glucan cross-linked with β-1,3-glucan covering 50–60% of the cell wall mass and hydrogen bonds to chitin crystalline [7,21]. Mannoproteins are glycoproteins composed of mannan polymers that bind to proteins via covalent bonds and are made up of 5–20% protein and 80–90% mannose linked by 1,6-α bonds that comprise around 40–50% of the yeast cell wall [22,23]. The mannoprotein layer is not only present on the surface but also in the deeper layers, protecting the glucan from external disturbances and creating a hydrophilic environment. Another dominant yeast cell wall compound is chitin, a linear polysaccharide composed of 1,4-2-acetamido-2-deoxy-b-D-glucopyranose. Chitin is an essential component of the cell wall, despite its presence in minute quantities in the cell residue (1–3% of the bulk of the cell wall) [24].

The rigidity and stability of the cell wall are attributable to β-1,3-glucan and chitin. Meanwhile, the covalent bond between the mannoprotein and the glucan layer contributes to the porosity of the cell wall, and it also contributes to the adhesion ability of yeast cells [25,26]. The glycoprotein component is firmly incorporated into the polysaccharide structure required for essential cellular processes such as maintaining cell morphology, protecting cells from foreign substances, transmitting intracellular signals, and synthesizing and remodeling other cell wall components [27]. The majority of yeast cell walls are hydrophilic, which limit the diffusion of hydrophobic molecules into the cell [28]. The components of the yeast cell walls also contribute to the mechanical strength of the cell, influence the encapsulated inner compounds, allowing polar and non-polar compounds up to 760 Da in molecular weight to diffuse freely [29].

β-glucan, mannoprotein, and chitin are the primary components of the cell wall in all species of yeasts, regardless of the type [30]. The cell wall components of *S. cerevisiae* can be regarded to represent nearly all *Ascomycetes*; however, the actual detailed composition is complex, with approximately 1200 genes playing a role in the cell wall [31]. Moreover, distinct yeasts species have distinct cell surface properties and cell wall compositions [32]. 

## 3. Lysis and Extraction of Yeast Cell Walls

To obtain β-glucan, mannoprotein, and chitin components for using them as polymers, pretreatment must be carried out for yeast cells. The process involves two main stages: yeast cell lysis and extraction of components from the cell wall. The cell wall contains β-glucan, mannoprotein, and chitin; hence, yeast cell lysis is needed to separate the cell wall from the cytoplasm before extracting the components [33]. 

**Figure 2:**
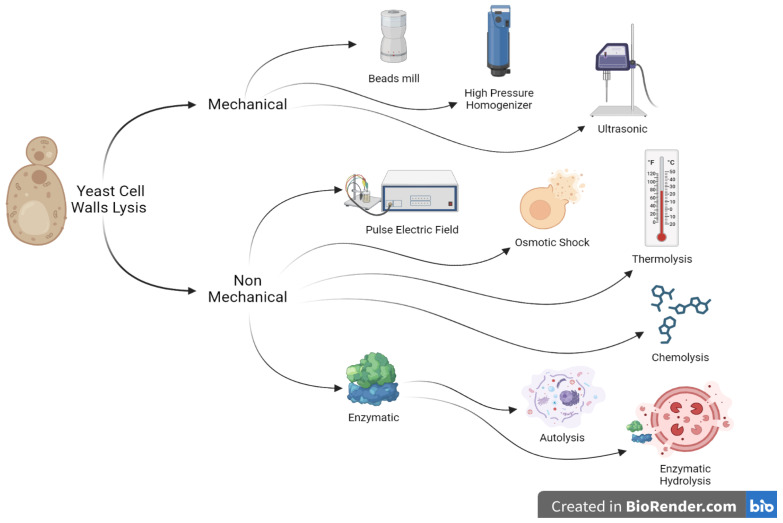
Yeast cell walls lysis methods.

As seen in Figure 2 yeast cell lysis methods can be classified into mechanical and non-mechanical methods (electrical, physical, chemical, and enzymatic). Mechanical methods are commonly used for large-scale industrial cell destruction, such as bead mills, high pressure homogenization (HPH), and ultrasonication (US) [34]. However, non-mechanical methods are more selective and gentler and are commonly used on a laboratory scale due to operational and economic limitations [35]. Bead mill is an effective mechanical method for destroying yeast cell walls, but it requires intensive cooling and energy. It also has the potential to destroy the protein sought, but its efficiency in destroying cells is limited by the influence of grains or paddles and increased costs in the purification process [36]. High-pressure homogenizers can be applied to damage yeast cells through increasing cell suspension pressure and then releasing it via a specially designed, adjustable valve assembly [37]. Ultrasonication is another method of mechanical cell damage that relies on the large shear forces created via high-frequency ultrasound [38].

Non-mechanical methods include electrical, enzymatic, chemical, and physical methods. Electric methods involve damaging cells by applying an electric field, while physical methods involve decompression, osmotic shock, and thermolysis. Pulsed electric field (PEF) is an efficient technique for destroying microbiological cells by controlling electrical parameters like voltage, treatment time, and energy [39]. Decompression occurs when pressurized gas causes disturbance to the cell, while osmotic shock and thermolysis involve diluted suspension of cells after reaching equilibrium under high osmotic pressure and heat treatment [40]. Chemical and biochemical methods involve using chemicals or enzymes during the cell disruption process. Yeast cells can be lysed using NaOH, HCl, acetic acid, citric acid, surfactants, detergents, or other aggressive chemical solutions. Enzymatic methods divide yeast cells into autolysis and hydrolysis processes [41].

Autolysis of yeast cells via endogenous enzymes is a degradation process triggered by the activation of yeasts intracellular enzymes. This process begins with the disorganization of biological membranes caused by cell death, leading to decreased activity of respiratory enzymes and accelerated activation of hydrolytic enzymes [42]. Glucanase and proteinase enzymes disrupt the cell wall, causing it to become porous and conical, triggering the release of intracellular compounds into the surrounding media [43]. The yeast cell wall constricts during autolysis, releasing large, medium, and tiny molecular weight proteins, long-chain and short-chain fatty acids, and polysaccharides through passive transfer [44]. The effects of intracellular enzymes, such as proteases and nucleases, also cause hydrolysis of intracellular compounds, allowing for the release of decomposition products [41].

In addition to autolysis, yeast cells also undergo lysis with certain lytic enzymes. Enzymatic hydrolysis can be carried out by either microbial enzymes or other exogenous cell wall proteolytic enzymes [45]. Enzymes are commercially available at affordable prices and the most efficient enzymes for yeast cell wall lysis include Zymolyase, Lysozyme, Glycosidase, Glucanase, Peptidase and Lipase [46]. 

After the lysis of yeast cell walls, the pretreatment method was continued for extracting the yeast cell wall components (Table 1). The yeast cell walls were dissolved in distilled water and centrifuged for recovery; then, it was washed three times and extracted twice with phosphoric acid. The insoluble residue was resuspended in distilled water and decanted with water until the pH reached 7 and resulted in 13.5% of β-glucan. Meanwhile, some methods can be combined to gain a higher yield and purity of β-glucan. The combination of thermolysis and sonication could yield up to 14% of β-glucan, while the improvement with protease and spray drying yields 11.2% of β-glucan from brewery spent with purity up to 93%.

In addition to β-glucan, mannoproteins can also be extracted through several methods. Laminarinase and sodium deodecyle sulfate were used in the process of isolating and extracting mannoproteins. The cells were washed twice and then digested with 1200 units of glucanase over the course of three hours and then centrifuged to obtain ±7.25% of mannoprotein. Hexadecyltrimethylammonium bromide can be used for precipitation and purification of the mannoprotein; then, the precipitate was dialyzed against deionized water for 48 h to improve the mannoprotein yield to 8.42%. Another biopolymer contained in yeast cell walls is chitin that can be extracted through centrifugation after chemolysis; the extracts were resuspended in HCl, neutralized with NaOH, dialysed, and lyophilized to obtain 2.4% of chitin.

### 3.1. β-Glucan Extraction

The first important step in the β-glucan extraction process is the preparation of the cell wall because β-glucan is located in it. For this reason, cell lysis or disruption is needed so that the cytoplasm will flow out and a pure cell wall is obtained. Extraction of β-glucan is carried out after the yeast cells have been lysed or damaged and the cytoplasm has been released [54]. Yeast cell lysis is achieved via physical (sonication and homogenization) and chemical (alkaline and acid) and/or enzymatic (lytic enzymes and glucanase) procedures. The mannoprotein compounds, and then the lipids and proteins are removed, resulting in different pure β-glucan fractions [55].

β-glucan from yeast cell walls were extracted using various extraction and purification methods. Enzymatic lysis of yeast cell walls releases soluble cytoplasm for centrifugation as supernatant without harsh chemicals [56]. Sonication was performed to disintegrate the yeast cell wall and extract the lipid and protein contents to obtain pure β-glucan [52]. Extraction β-glucan also can be carried out from spent brewer’s yeasts which is a by-product of alcoholic fermentation [57]. 

### 3.2. Mannoprotein Extraction

Mannoprotein is the yeast cell wall’s second most essential component (40%, *w*/*w*) that consists of glycoprotein with 50–95% polysaccharides [58]. Yeast protein is rich in the important amino acids lysine, tryptophan, and cysteine, making it more nutritious than other vegetable proteins [59]. Mannoproteins can be non-covalently bound, covalently bound, or disulfide bonded to cell wall proteins that are covalently bound to glucan structures. Alkaline-sensitive cell wall proteins (Pir-CWPs) and glycosylphosphati-dylinositol-CWPs (GPI-CWPs) are covalently bonded mannoproteins, with GPI CWPs as the main class of yeast cell walls protein [60].

The extraction of mannoprotein with heat treatment using sodium dodecyl sulfate yielded lower amounts than enzymatic treatment without glycosylated protein [59]. The enzymatic method using the Zymolyase enzyme produced the highest yield, the highest mannoprotein content, and the highest ratio of mannan to protein. The enzymatic method with the Zymolyase enzyme relies on the hydrolytic activity of β-glucanase to hydrolyze β-glucan, opening the yeast cell wall structure and then effectively releasing the mannoprotein [61].

### 3.3. Chitin Extraction

Chitin is a large polysaccharide with nitrogen crystals made of N-acetyl-D-glucosamine. As the second most prevalent polysaccharide after cellulose, chitin is the major structural component of fungal, insect, and crustacean exoskeletons [62]. β-(1,4)-linked D-N-acetylglucosamine (GlcN) and N-glucosamine (Glc-NAc) units form a linear biopolymer of chitin. Partial deacetylation of N-acetyl-D-glucosamine to D-glucosamine converts chitin into chitosan [63].

Unlike cellulose, which has been extensively studied, chitin is underutilized despite its abundance and possible future uses. High-purity chitin requires removal of certain contaminants such as proteins and minerals that bind to biomass chitin. The extraction of chitin from the yeast cell wall can be performed through mechanical lysis of yeast cells using a homogenizer, followed by alkaline and acid extraction [64].

## 4. Separation and Purification of Yeast Cell Walls

The important biopolymers found in yeast cell walls, such as mannoprotein, chitin, and β-glucan, have a wide range of uses in a variety of fields, including food, medicine, and nanotechnology. Techniques for separation and purification are necessary in order to effectively utilize these biopolymers. Some separation and purification methods destroyed and disseminated yeast cell walls biopolymers such as β-D-glucans in supernatant which significantly affected its biological function [49].

Some methods used in the separation and purification of yeast cell wall biopolymers are presented on Table 2. It can be seen that diethylaminoethyl (DEAE) anion exchange chromatography is a better method for separating and purifying yeast cell wall biopolymers, resulting in a higher yield of β-glucan, while concanavalin-A chromatography could be an alternative to increase the purity based on the resulting glucan/mannan ratio. Meanwhile, affinity chromatography using a HiTrap Con A 4B column separated mannoproteins from non-glycosylated proteins [65]. The use of Zymolyase was one of the approaches that was combined with others. Mannoproteins that were recovered following Zymolyase^®^ treatment were purified on a Concanavalin-A sepharose affinity chromatography column utilizing a KTA purifier system (GE Healthcare, USA) [66]. These methods were also followed by dialysis to improve the purity of chitin [67].

## 5. Compatibility of Encapsulant Biopolymers from Yeast Cell Walls

Selection of suitable encapsulant materials for probiotic cells is very important to consider in order to maintain the stability and properties of the resulting products. After the encapsulation process, the coating material will be in direct contact with the host’s digestive tract and numerous environmental factors that threaten it. For these reasons, many general criteria have been developed for selecting the appropriate encapsulant material. When choosing encapsulant materials for probiotic encapsulation, several factors must be considered: (a) physicochemical properties (chemical composition, morphology, mechanical strength, and stability in the gastrointestinal tract); (b) toxicology test; (c) manufacturing and sterilization processes; (d) release mechanism; and (e) functional properties of the ingredients in the final product [13,70]. Due to lipid membrane oxidation, temperature and moisture can impair probiotic cell viability during storage; the encapsulated cells will survive better using moisture-retaining materials [71].

As mentioned in Table 3, yeast cell wall biopolymers have some potential characteristics. Regarding psychochemical properties, β-glucan from yeast cell walls resembles a honeycomb structure exhibiting mechanical strength, while chitosan has better protection ability due to its great molecular weight [72,73]. Thermal stability of the yeast cell walls was shown up to 265 °C, and the β-glucan encapsulant exhibited less sensitivity toward heat treatments [6,74]. β-glucan could also play the role as a cryoprotectant that maintains probiotics viability and strengthens cell protection and attachment [14,75,76].

All the yeast cell wall biopolymers were non-toxic substances and recognized as GRAS, while chitin has been found to have very good biocompatibility and biodegradability as well as a characteristic cationic action [2,6]. Usefulness of yeast cell wall biopolymers were proven as mannoprotein demonstrated its ability to inhibit bacterial colonization of the gut and regulate adhesion, while chitin demonstrated its ability to interact with other molecules to enhance its biological activities [17,26,77,78].

**Table 3:** Properties of yeast cell wall biopolymers as probiotic encapsulants.

Characteristics	Description	References
Psychochemical properties		
*Mechanical strength*	- The structure of β-glucan resembles a honeycomb, which is why probiotic cells are trapped more easily.	[72]
	- Chitosan, with a greater molecular weight, creates a microcapsule membrane that is thicker and thus better protects the probiotic bacteria encapsulated within.	[73]
*Thermal stability*	- The thermal stability of yeast cells is advantageous for employing yeasts as microcapsules, as yeast cells are stable up to 265 °C.	[6]
	- Encapsulated probiotic cells with β-glucan survived in 55 °C for 10 min; however, when the temperature increased, they were less sensitive to heat than free probiotic cells. When heated from 55 °C to 75 °C for 10 min, free probiotics of 7.01 to 1.01 log CFU/g, 6.15 to 0.96, and 6.15 to 0.99, were yielded, respectively.	[74]
*Cryoprotectant*	- β-glucan extracted from *S. uvarum* spent brewer’s yeasts is a good cryoprotectant for 120-day freeze-dried probiotic cultures, with a lower viability decline (1–3 log cycles) than saline solution (4 log cycles). All strains of β-glucan maintained probiotic levels for over 60 days.	[14]
*Protection*	- β-glucan offers protection through high polymerization, hydroxyl group presence, and cell attachment.	[75,76]
Toxicology		
*Non-toxic*	- Chitin is non-toxic, possesses distinctive cationic properties, and is highly biocompatible and biodegradable.	[2]
*Generally recognized as safe*	- Yeast cells are excellent and novel potential encapsulation materials for the food industry because of their GRAS status, nature, and low cost.	[6,11]
Functional properties		
*Anti-pathogenic*	- Mannoprotein reduces enteropathogenic bacteria colonization to the intestine by binding lectins to D-mannose receptors.	[77]
*Adhesion*	- Mannoprotein affects the porosity of the cell wall to regulate the entry and exit of macromolecules into and out of the environment, which has a profound effect on the adhesion ability of microbes.	[26]
	- Acid hydrolysis of thermally extracted mannoproteins reduced adherence ability to Caco-2 cells.	[79]
*Functionality*	- Chitin’s amino group protonation increases solubility, allowing to interact with other molecules, and exhibits various biological effects, including antibacterial, antifungal, anti-inflammatory, and cancer activities.	[78]

The encapsulation is performed using a microcapsule that is thin, semi-permeable or non-permeable membrane around a solid or liquid core of up to 1 mm diameter. Alginate, chitosan, carboxymethyl cellulose, xanthan gum, starch, carrageenan, gelatin, pectin, dairy products including casein and milk whey, and other food-grade biopolymers have been used in various encapsulation procedures [80]. Spray drying, freeze-drying, fluidized bed coating, extrusion, emulsification, conservation, and electrostatic methods are well-known microencapsulation techniques that produce capsule outputs of 10–400, 20–5000, 5–5000, 150–8000, 10–1000, and 10–800 μm, respectively [81].

Yeast cells were recognized as GRAS material, nature, low cost, health benefits, and thermal non-degradability which make the cell an excellent and novel potential encapsulation material for the food industry [6,10,11,13]. Yeast cells have been successfully applied to encapsulate flavor substances; it is also known that encapsulation in yeast cells can increase active thermo/oxidative stability which exceeds the effect of commonly used coating agents such as maltodextrin or cyclodextrin [6,9,16,82,83]. 

Yeast cell walls also can be employed as encapsulant materials due to their constituent components which highlight their potential as good coating materials. The cell wall of *S. cerevisiae* consists of a 10 nm long layer of highly glycosylated mannoprotein, a network of 1,3 β-glucan, and 1,6 β-glucan connected to the underlying chitin film. β-glucan and chitin provide cell rigidity properties, while chitin independently has a very large tensile strength, and plays a role in maintaining the structural integrity of the cell wall [6]. The mannoprotein component is tightly integrated in the polysaccharide structure, and it maintains cell shape, provides cell porosity, protects cells from foreign substances, transmits intracellular signals, and synthesizes and overhauls other cell wall components [12].

### 5.1. β-Glucan as a Probiotic Encapsulant

Because of its unique honeycomb-like structure, unlike other polysaccharides, β-glucan can be employed as an encapsulant. Probiotics are easily trapped in tissue structures due to this peculiarity [84]. Valorization of β-glucan extracted from barley to encapsulate probiotic bacteria *L. plantarum*, *L. casei* and *L. brevis* with emulsification techniques showed significant improvement in tolerance toward stresses like low pH, heat treatment, simulated intestinal conditions and storage [74]. 

Scanning electron microscopy (SEM) measurements demonstrate that β-glucan has an intact cell wall structure resembling a honeycomb with a rough surface and large hole diameters [85]. The honeycomb-like internal structure and anti-cracking and compact surface features may help the β-glucan to resist mechanical stress and shield trapped components from hostile conditions including oxidation, light, low or high pH [86]. SEM micrographs of β-glucan’s application as a probiotic encapsulant show that several bacterial cells were randomly distributed throughout the matrix [74]. Microencapsulation using the freeze-drying technique caused the structure of the β-d-glucan walls to partially collapse and the bacterial cells were randomly distributed in the matrix [72,87]. The thermophysical characteristics of β-glucan have been show have wide water-related endothermic peaks between 50 and 120 °C; meanwhile, the endothermic and exothermic peaks over 250 °C and 350 °C are thought to be related to the thermal degradation of the β-glucan matrix [74]. 

Using β-glucan from yeast cell walls as an encapsulant is advantageous since yeast cells are stable up to 265 °C, beyond which their cell walls begin to breakdown [13]. The yeasts at temperatures of 25–110 °C experienced a slight loss of mass and at 110–263 °C, the mass remains constant; meanwhile, raising the temperature to about 263–293 °C causes the cell walls to break down. Finally, at higher temperatures (293–400 °C), the dry constituents of the cell are unstable, and eventually carbonize [88].

Encapsulated probiotic cells survived at 55 °C for 10 min; however, when the temperature increased, they were less sensitive to heat than free probiotic cells. The thermal tolerance of *L. casei*, *L. acidophilus* and *B. bifidum* cells encapsulated with β-glucan and heated at 60 °C for 10 min increased by 23.04–45.33%, 32.72–39.19% and 23.20–50.35% respectively [74]. β-glucan can protect cells in its matrix from hazardous external circumstances [89]. β-glucan from spent brewer’s yeast, *Saccharomyces uvarum*, can protect probiotic bacteria *L. acidophilus* and *L. plantarum* better than fructooligosaccharides/FOS. β-glucan and FOS are good cryoprotectants for 120-day freeze-dried probiotic cultures, with a lower viability decline (1–3 log cycles) than saline solution (4 log cycles) [14]. 

β-glucan and fructooligosaccharide are cryoprotectants with similar environmental protection abilities due to their genotype and cell wall composition [90]. The protection provided by β-glucan is thought to be related to the degree of polymerization of β-glucan, which is high (>100), as well as the presence of several hydroxyl groups in its structure [75]. Hydroxyl groups restore water and stabilize the membrane, keeping the biological structure in the same state as before freeze-drying [76]. Carbohydrate polymers are the best protectors because they allow probiotic cells to be accommodated between the space and the surface of the biopolymer molecule arrangement, similar to the honeycomb-like structure of β-glucan, so the probiotic cells are trapped more easily [72].

### 5.2. Mannoprotein as a Probiotic Encapsulant

Mannoproteins play an important role in supporting the use of yeast cells as encapsulants. The inner plasma membrane and the rigid structure of yeast cells make yeast cells a suitable encapsulant material, where mannoprotein contributes to porosity during encapsulation [6]. Mannoproteins, highly glycosylated glycoproteins in the yeast cell wall’s outer layer, are covalently linked to the amorphous β-1,3-glucan matrix. Mannoprotein’s covalent connection with β-glucan regulates the yeast cell wall’s porosity, regulating macromolecule entry and exit and the cell adherence [26,91,92]. 

Adhesion to intestinal cells is a desirable property for probiotics in the gastrointestinal tract because of their potential to provide health benefits to the gut. The mannoprotein extraction method and mannose availability in the fraction affected the extract’s efficacy of bacteria cell adhesion and invasion against Caco-2 cells. Mannoproteins derived from thermally extracted yeasts had the highest capacity to inhibit Caco-2 cell invasion; meanwhile, mannoproteins derived from thermally extracted yeasts that undergo acid hydrolysis decrease the adhesion to Caco-2 cells, which confirmed the increase in active mannose monomers presence that compete for adhesion [79]. Mannose-based carbohydrates can also inhibit enteropathogenic bacteria colonization because bacterial lectins bind to receptors containing D-mannose [77].

The application of mannoprotein extract in lactic acid bacteria significantly increased the adhesion of *L. plantarum*, *L. salivarius*, and *E. faecium* to Caco-2 cells but decreased the adhesion of *L. casei* and *P. damnosus*, suggesting that the protein moiety of the mannoprotein is important [17]. The antiadhesive properties of mannoproteins are associated with the mannose fraction because the attachment of bacteria to intestinal cells is influenced by the binding of bacterial lectins to cell receptors containing D-mannose [77]. The adhesion of *L. plantarum* and *L. acidophilus* was strongly inhibited by the presence of D-mannose, several proteins on the cell surface are also thought to be involved in this [93,94].

### 5.3. Chitin as a Probiotic Encapsulant

Chitin is a natural polymer found in fungi, green algae, insect cuticles, crab and shrimp shells, and yeast cell walls, while chitosan is exclusively present in specific fungi [95]. Chitosan, a commercially relevant biopolymer, can be obtained from chitin through a partial N deacetylation process at temperatures between 60 and 80 °C using 50% *w*/*w* alkali [96]. Deacetylation and molar mass affect chitosan’s properties and usage. Chitosan with a greater molecular weight creates a thicker microcapsule membrane and preserves probiotic bacteria better than free cells and low-molecular weight chitosan [73]. Homogeneous or heterogeneous deacetylation conditions affect chitosan microstructure, solubility, and drug or food delivery [97].

Deacetylation determines the number of free amino groups in chitosan, making it soluble in diluted acid solutions [98]. The presence of amino groups that form a positively charged surface, and hydroxyl groups which represent a negative charge, determines the functionality of chitosan. The amino group can be easily protonated and it increases the solubility of chitosan in aqueous acid solutions. Thus, chitosan can interact with other molecules in solution and exhibit broad biological effects, including antibacterial, antifungal, anti-inflammatory and anti-cancer activities, as well as fat binding, film forming, antioxidant and chelating capacities; hence, chitosan is widely used [78]. In addition, the main advantages of chitosan as an encapsulant are its unique cationic character, high biocompatibility and biodegradability, and non-toxicity; thus, it has received much attention as a potential encapsulant that can be used in probiotic microencapsulation [2].

There are problems with chitosan when used as a coating, where chitosan shows an inhibitory effect on bacteria such as *L. lactis* [99]. However, due to its cationic nature and ability to survive in acidic media, chitosan remains the most widely used coating material for protecting probiotics, which are widely used to coat other negatively charged coatings to strengthen microcapsules, but not the capsule itself [19].

Chitosan has been found formulated with alginate when it is applied as a probiotics encapsulant due to some disadvantages of alginate, such as sensibility that can be decomposed under acidic conditions [100,101,102]. Formulating the alginate beads with chitosan forms a complexation reaction and produces beneficial properties for probiotics encapsulation, such as reduced porosity of the alginate beads, reduced leakage in the microcapsule, stability over various pH ranges, and reduced oxygen exposure to probiotics, thus increasing their stability under unfavorable conditions [103]. The nature of the negative charge of the alginate in contact with the positive charge of chitosan with ionic bonds forms a semipermeable membrane; therefore, the resulting capsule has a smoother surface with reduced permeability to water-soluble molecules [104].

## 6. Role of Yeast Cell Wall Biopolymers as Encapsulants in Protecting Probiotics 

Some applications of yeast cell wall biopolymers as probiotic encapsulants have been presented (Table 4). Encapsulation of the yeast cell wall and use of calcium alginate to wrap the microbeads of probiotics bacteria resulted in protective ability that delivers viable probiotics until the colon and especially boosted *L. acidophilus* tolerance toward acid. After mixing probiotics with yeast cell walls in a saline solution optimized with agitation and an orbital agitator for encapsulation, probiotic survival is shown to increase. Meanwhile, the formulation of β-glucan from yeasts mixed with cell suspensions and lyophilized showed similar protective activities with FOS that could also act as potential prebiotics. Other studies have demonstrated chitosan’s potential to increase resistance to simulated gastric and intestinal fluids. Even though some optimization and combination of methodology are still needed, some applications of yeast cell wall biopolymers as encapsulants have shown some potential. 

Apart from using intact yeast cells in encapsulation, the yeast cell wall itself has the potential to be used as a microcapsule. Some application utilized the yeast cell walls through physical disintegration and bigger probiotic bacteria were encapsulated in it. Probiotic bacteria *L. acidophilus* and *B. bifidum* were encapsulated with three coating layers, an emulsified calcium alginate. *S. cerevisiae* cell walls re-coated with calcium alginate layer promote probiotic bacteria survival in simulated gastric juice by 4–7% with the viability of more than 10^7^ cfu/g after 2 h simulation; meanwhile, free probiotic bacteria had the biggest log reduction [10]. *L. acidophilus* coated with *S. cerevisiae* cell wall was more GIT-resistant than other treatments. The yeast cell wall slows gastric juice absorption into the microcapsule. However, for *B. bifidum*, the log reduction values for single, double and triple layer microcapsules were not significantly different, especially when the pH is below 1.55, because they are acid-sensitive and need more protection [105].

The efficiency of β-glucan in encapsulating probiotic bacteria *Lactobacillus casei*, *Lactobacillus brevis*, and *Lactobacillus plantarum* is simulated in gastrointestinal conditions [74]. The stability of β-glucan at low pH and the lack of hydrolyzing enzymes in the frontal digestive system confirm these results [106]. Better cell viability due to endogenous production of β-glucan by *L. paracasei* in simulated intestinal juice was also reported [89]. β-glucan supports cell wall resilience by preventing cells from interacting with bile salts, the characteristics of which is commonly found in oligosaccharides-based encapsulants [107,108]. When directly exposed to the simulated intestinal juice, encapsulated bacterial cells were released from the β-d-glucan matrix after 25 min, which indicated that β-d-glucan provides protection against probiotics because its stability at low pH can also prevent earlier bacteria release [109].

Swelling index (%) is an important parameter that determines the ability of the encapsulation matrix to sustain probiotic cells during transit through the GI tract. The higher the swelling index value, the greater the resistance offered by the encapsulation material to the coated cells so that the release time can be delayed [110]. β-d-glucan consisting of β-(1,4)-bonds is flexible and capable of inter-chain aggregation through the formation of junction zones, thereby reducing solubility [111]. The swelling index of β-d-glucan microcapsules was highest at stomach pH 3 and 4; meanwhile, at pH 6.5, which is the small intestine’s pH range, the β-d-glucan microcapsules disintegrated with the decrease in the swelling index [72]. It can be concluded that the release of probiotic cells from the yeast cell wall matrix as an encapsulant is dependent on pH and the microcapsules begin to dissolve when they enter the small intestine where probiotics are most needed [112].

**Table 4:** Role of yeast cell wall biopolymers as probiotic encapsulants.

Probiotics	Formula	Results	References
*L. acidophilus* and *B. bifidum*	Probiotic bacteria were encapsulated with calcium alginate using the emulsion method; then, the microbeads were covered by the *S. cerevisiae* cell wall and then re-encapsulated with the final layer of calcium alginate.	*S. cerevisiae* cell wall compounds provide a protective barrier for delivering viable bacterial cells to the colon. They improve acid tolerance for *L. acidophilus* but not for *B. bifidum*, making their protective ability dependent on microbe type.	[10]
*L. acidophilus* LA-05, *L. plantarum* 49, and *L. plantarum* 201	β-glucan from yeasts mixed with cell suspensions were kept for 1 h at room temperature. The suspensions were divided into 1 mL aliquots, transferred aseptically into 5 mL containers, and frozen at 20 °C for 24 h. The samples were freeze-dried in a benchtop lyophiliser for 40 h at 55 2 °C and 1 mm/h. After freeze-drying, the containers were sealed and refrigerated for 120 days at 0.5 °C.	β-glucan is a potential cryoprotectant for probiotic lactobacilli, providing similar protection to fructooligosaccharides after freeze-drying, storage, and exposure to simulated gastrointestinal conditions. It offers potential applications as a functional food ingredient and can be obtained from by-products of the beer industry, which reduces environmental impacts.	[14]
*L. acidophilus*	*L. acidophilus* were dissolved in saline and mixed with yeast cell walls, agitated on an orbital shaker, and optimized for encapsulation. Filtration was improved with vacuum-filtered glass funnels and filter holders; then, the filtrate was centrifuged at 5000 rpm for 15 min.	The viability of the encapsulated cells was 19.048 ± 2.701%, while the majority of free cells could not survive 150 min of treatment with SGJ at pH 2. Encapsulated *L. acidophilus* were enhanced, with greater survival at 56.338 5.094%.	[113]
*S. boulardii*	Briefly, 1 g of chitosan in 100 mL of distilled water is acidified with 0.4 mL glacial acetic acid to 3.6. Chitosan solution was autoclaved (121 °C for 15 min) before use. A magnetic bar swirled alginate particles in chitosan solution for 30 min. Probiotic cells were suspended, filtered, and rinsed with distilled water.	Low-cost external ionic gelation and drying at 40 °C maintain *S. boulardii* survival, with chitosan coating providing increased resistance to yeasts and protection against simulated gastric and intestinal fluids.	[101]

β-glucan extracted from spent brewer’s yeasts was useful as a good cryoprotectant for freeze-dried probiotic lactobacilli bacteria when exposed to simulated gastrointestinal conditions [72]. The control strain, hydrated in saline after freeze-drying, exhibited a higher decrease in viability during in vitro digestion (up to 2 log cycles) than freeze-dried bacterial samples that were encapsulated by β-glucan (0–1 log cycles) [14]. β-glucan also has the potential as a prebiotic that can encourage the growth of beneficial microflora in the digestive tract and can protect probiotics during product manufacturing [114]. The presence of β-glucan as prebiotics during the growth of probiotics strains can increase the resistance of probiotics bacteria against conditions of the digestive tract.

Mannoprotein derived from yeasts were also shown to increase the growth and viability of probiotic bacteria in simulation. As a carbon source, the mannoprotein extract was more effective than the whole yeast cell wall when metabolized by probiotic bacteria that could increase their growth [17]. The protective function of the mannoprotein extract and yeast cell wall is thought to correlate with the effect of bacterial growth which are also stimulated by the presence of this extract.

Another yeast cell wall component is chitin that can be converted to chitosan. Chitosan can work as a buffer by reducing encapsulant permeability under acidic conditions so that its impact on probiotic bacteria viability can be avoided, whilst maintaining its integrity by reducing probiotic bacteria shedding [108]. Cationic properties and stability in acidic media has been found in chitosan when used as an encapsulant material in microcapsules which results in the resistance against gastric juice [115,116]. In simulated gastrointestinal circumstances, chitosan encapsulation enhanced *Lactobacillus reuteri* DSM 17938 survival [117]. Another encapsulation method with oppositely charged polyelectrolytes between chitosan and dextran sulfate resulted in a strong electrostatic interaction that forms a compact structure protecting the probiotic that generates high viability [115]. Meanwhile, the encapsulant formulation of chitosan–alginate showed some promising results in simulated gastric juice environment such as no release of *Enterococcus faecium* cells until 144 h of encapsulation; there are increases in probiotic bacteria viability after 2 h and the number of probiotic is maintained above 10^7^ cfu/mL [118,119]. 

## 7. Conclusions

There is a lot of potential for the yeast cell wall to be used as a probiotic encapsulant material. The yeast cell wall is mostly made up of β-glucan, mannoprotein, and chitin. The structural integrity and durability of the cell wall can be attributed, in part, to the presence of β-glucans, mannoproteins, and chitin all working together. Because of its excellent thermal stability, β-glucan has proven to be an efficient cryoprotectant for probiotic bacteria throughout the freeze-drying process. It has been demonstrated that the utilization of mannoprotein can improve the adhesion capabilities of probiotic bacteria on the epithelial cells of the intestine. Chitosan, which is obtained from chitin via a process known as deacetylation, is frequently utilized in a wide variety of industrial applications due to the advantageous properties that it possesses. It has been proven that the presence of β-glucan, mannoprotein, and chitosan has a key effect in the protection of probiotic microbes within the gastrointestinal system. Additionally, both β-glucan and mannoprotein possess prebiotic qualities, and when combined with probiotics, these ingredients can result in the production of synbiotic compositions. Their viability as novel coating materials for probiotics is further strengthened by the functional properties they possess, and thus, they find applications in the food sector.

Future possibilities for the use of yeast cell walls in the food industry are quite promising, as they could lead to improved food functioning, health advantages, and sustainability. However, issues with extraction, regulatory approval, cost, and consumer acceptance must be resolved in order to make their commercialization successful. The food industry can unlock the enormous potential of yeast cell wall derivatives and change the manufacturing of useful and healthier food products by supporting research, adopting sustainability principles, using biotechnology, and increasing consumer awareness.

## Figures and Tables

**Table 1:** Processes of yeast cell walls extraction.

Resources	Extraction Processes	Results	References
Yeast cell walls *S.cereviseae*	The yeast cell walls were extracted with NaOH, dissolved in distilled water, and collected via centrifugation. The insoluble material was recovered, washed three times, and extracted twice with phosphoric acid. The insoluble residue, representing cell wall β-d-glucan, was separated, resuspended in distilled water, and decanted with water until pH 7.	(1→3)-β-d-glucan with yield 13.5%	[47]
*S. cerevisiae*	Yeast cell exposure to hot water (autoclaving), thermally induced autolysis, homogenization in a bead mill, sonication and their combinations.	13–14% of β(1,3)/(1,6)-glucans	[48]
Spent brewer’s yeast (*S. cerevisiae*) slurry, a brewery by-product with 18% solids	Preliminary purification, induced autolysis, hot water treatment, homogenization, organic solvent treatment, protease treatment, and spray drying.	β-d-glucan, with 93% purity and 11.2% yield	[49]
*S. cerevisiae* K48L3, K48L4, YPH499	Mannoproteins were isolated and extracted using SDS and laminarinase. The late logarithmic phase cells were harvested, washed twice, and digested with 1200 units of glucanase for 3 h. The extract was centrifuged, and glucanase-extracted mannoproteins were purified using ion exchange and affinity chromatographies.	725–2255 µg mannoprotein/100 mg dry weight of yeasts	[50]
*K. marxianus*	The yeast cell precipitate was re-suspended in a buffer solution, washed with acetic acid, and precipitated. The supernatant was incubated overnight and centrifuged. Hexadecyltrimethylammonium bromide was used for selective precipitation and purification of the mannoprotein. The precipitate was then dialyzed against deionized water for 48 h.	8.42 ± 0.06% crude mannoprotein	[51]
*S. uvarum*	The insoluble material from autolyzed brewer’s yeasts slurry was diluted, heated, and washed three times with distilled water. Sonication, lipid extraction, and proteolysis were performed, and the insoluble residue was washed five times. Mannoprotein was precipitated, washed, dialyzed, and lyophilized.	4.16% yield of mannoprotein	[52]
*S. cerevisiae* W301-1A	Cell walls were lyophilized and subjected to alkaline and acidic extractions. After centrifugation, the extracts were collected and used for subsequent steps. The samples were then resuspended in HCl, neutralized with NaOH, dialysed, and lyophilized.	2.4% of chitin	[53]

**Table 2:** Methods for separation and purification of yeast cell wall biopolymers.

Strain	Methods	Yield	Other Results	References
*S. cereviseae*	DEAE chromatography	10.36% β-glucan	Protein (0.004%) Carbohydrate (0.090%) Glucose (0.022%) Mannose (0.069%)	[34]
*S. cereviseae*	DEAE chromatography	13.00% β-glucan	Protein (0.3%) Glucan/Mannan ratio (30/70)	[68]
*S. cereviseae*	Concanavalin-A chromatography	0.32% β-glucan	Glucose (0.014%) Mannose (0.000%)	[34]
*S. cereviseae*	Concanavalin-A chromatography	4.00% β-glucan	Glucan/Mannan ratio (100/0)	[68]
Baker’s yeast *S. cereviseae*	SDS extraction followed by Concanavalin-A chromatography	0.98% Mannoprotein	Mannan/Protein ratio (31/100)	[59]
*C.albicans*	Mercaptoethanol and sodium dodecyl sulfate followed by Concanavalin-A chromatography	1.5mg/13g Mannoprotein	30–55 kDa	[69]
*Saccharomyces cerevisiae*	Zymolase followed by Concanavalin-A chromatography and dialysis	127.4 ± 3.2 μg/mg β-glucan 6.2 ± 0.55% Chitin	Mannan (93.3 ± 3.2 μg/mg)	[67]

## Data Availability

Not applicable.
